# Mortality variability and differentials by age and causes of death in rural South Africa, 1994–2018

**DOI:** 10.1136/bmjgh-2023-013539

**Published:** 2024-04-08

**Authors:** Brian Houle, Chodziwadziwa Kabudula, Sanyu A Mojola, Nicole Angotti, Francesc Xavier Gómez-Olivé, Dickman Gareta, Kobus Herbst, Samuel J Clark, Jane Menken, Vladimir Canudas-Romo

**Affiliations:** 1 School of Demography, The Australian National University, Acton, Australian Capital Territory, Australia; 2 MRC/Wits Rural Public Health and Health Transitions Research Unit (Agincourt), Faculty of Health Sciences, School of Public Health, University of the Witwatersrand, Johannesburg, South Africa; 3 Institute of Behavioral Science, University of Colorado Boulder, Boulder, Colorado, USA; 4 Department of Sociology, School of Public and International Affairs, and Office of Population Research, Princeton University, Princeton, New Jersey, USA; 5 Department of Sociology, American University, Washington, DC, USA; 6 Africa Health Research Institute, Durban, KwaZulu-Natal, South Africa; 7 DSI-MRC South African Population Research Infrastructure Network, Durban, South Africa; 8 Department of Sociology, The Ohio State University, Columbus, Ohio, USA

**Keywords:** HIV, AIDS, Child health, Epidemiology, Public Health

## Abstract

**Introduction:**

Understanding mortality variability by age and cause is critical to identifying intervention and prevention actions to support disadvantaged populations. We assessed mortality changes in two rural South African populations over 25 years covering pre-AIDS and peak AIDS epidemic and subsequent antiretroviral therapy (ART) availability.

**Methods:**

Using population surveillance data from the Agincourt Health and Socio-Demographic Surveillance System (AHDSS; 1994–2018) and Africa Health Research Institute (AHRI; 2000–2018) for 5-year periods, we calculated life expectancy from birth to age 85, mortality age distributions and variation, and life-years lost (LYL) decomposed into four cause-of-death groups.

**Results:**

The AIDS epidemic shifted the age-at-death distribution to younger ages and increased LYL. For AHDSS, between 1994–1998 and 1999–2003 LYL increased for females from 13.6 years (95% CI 12.7 to 14.4) to 22.1 (95% CI 21.2 to 23.0) and for males from 19.9 (95% CI 18.8 to 20.8) to 27.1 (95% CI 26.2 to 28.0). AHRI LYL in 2000–2003 was extremely high (females=40.7 years (95% CI 39.8 to 41.5), males=44.8 years (95% CI 44.1 to 45.5)). Subsequent widespread ART availability reduced LYL (2014–2018) for women (AHDSS=15.7 (95% CI 15.0 to 16.3); AHRI=22.4 (95% CI 21.7 to 23.1)) and men (AHDSS=21.2 (95% CI 20.5 to 22.0); AHRI=27.4 (95% CI 26.7 to 28.2)), primarily due to reduced HIV/AIDS/TB deaths in mid-life and other communicable disease deaths in children. External causes increased as a proportion of LYL for men (2014–2018: AHRI=25%, AHDSS=17%). The share of AHDSS LYL 2014–2018 due to non-communicable diseases exceeded pre-HIV levels: females=43%; males=40%.

**Conclusions:**

Our findings highlight shifting burdens in cause-specific LYL and persistent mortality differentials in two populations experiencing complex epidemiological transitions. Results show high contributions of child deaths to LYL at the height of the AIDS epidemic. Reductions in LYL were primarily driven by lowered HIV/AIDS/TB and other communicable disease mortality during the ART periods. LYL differentials persist despite widespread ART availability, highlighting the contributions of other communicable diseases in children, HIV/AIDS/TB and external causes in mid-life and non-communicable diseases in older ages.

**WHAT IS ALREADY KNOWN ON THIS TOPIC**

- Studies of mortality burden and differences from the AIDS epidemic largely focus on cross-national comparisons using complex modelling, as well as clinical cohort studies reporting life expectancy gains among those living with HIV and on antiretroviral therapy.
- Other studies provide population-level estimates of life expectancy changes in HIV settings, however, these studies focus on contributions of causes of death to life expectancy differences and do not quantify the burden of mortality due to different causes of death at different periods of time.

**WHAT THIS STUDY ADDS**

- Our results highlight the contribution of child deaths to life-years lost during peak epidemic years and that most child life-years lost were not due to HIV/AIDS/TB.
- Life-years lost due to HIV/AIDS/TB and other communicable diseases has declined dramatically in both settings as treatment availability increased, but persistent differentials remain while emerging trends are shifting towards life-years lost due to non-communicable diseases and for men, external causes of death as an increasing proportion of all life-years lost.

**HOW THIS STUDY MIGHT AFFECT RESEARCH, PRACTICE OR POLICY**

- The convergence of high burdens of chronic infectious and non-communicable disease epidemics in resource-poor settings highlights the continued need for integrated and targeted public health programmes to support linked care for HIV/AIDS and concurrent treatment of non-communicable diseases.

## Introduction

Despite substantial progress in reducing AIDS-related deaths, the HIV pandemic continues to be a key driver of premature mortality—with over 39 million AIDS-related deaths to date.^1^ In sub-Saharan Africa, persistent and emerging health differentials due to HIV, other communicable and non-communicable diseases (NCDs) highlight the unfinished agenda of sustainable health for all from the Sustainable Development Goals.^2^ Beyond measures of life expectancy at birth, an expanding literature has highlighted variability in ages-at-death and the importance of examining mortality differences between and within countries.^3^ Monitoring changes in survival improvements has critical implications for how public health achieves continued progress in reducing avoidable premature deaths.

Recent evidence indicates substantial mortality heterogeneity in ages-at-death in populations undergoing dynamic epidemiological transitions,^4 5^ as well as within-country variability.^3^ However, information on mortality differences in many sub-Saharan African settings is limited, particularly in rural areas, given the need for complex information systems which comprehensively cover vital events with sufficiently large samples.^6^ Understanding mortality heterogeneity in sub-Saharan Africa is critically important given marked differentials in health risks and outcomes, including among people in rural areas.^2^

South Africa is an important setting in which to understand mortality variability given its complex and evolving epidemiological and health transition over the past two decades. Mortality dramatically increased from the mid-1990s to mid-2000s due to the AIDS epidemic.^7^ Subsequently, widespread introduction of antiretroviral therapy (ART) in public health facilities has resulted in substantial declines in AIDS-related mortality.^7 8^ However, the legacy of the apartheid era has resulted in persistent variability and uneven progress, particularly within rural regions. Rapid social and economic development has also altered the exposure to risk factors for NCDs and external injuries.

We assess changes in life expectancy, age distributions of mortality and cause-specific life-years lost (LYL) using longitudinal population surveillance data from two poor, rural South African populations over a 25-year period. The time periods examined include the changing impact of the AIDS and NCD epidemics, the roll-out of ART and rapid social change in the postapartheid era.

## Methods

### Setting and data

We used data on vital events and verbal autopsies (VA) from two sites in rural South Africa^9^: (1) the Agincourt Health and Socio-Demographic Surveillance System (AHDSS)^10^ in Agincourt subdistrict in rural Mpumalanga Province and (2) the Africa Health Research Institute (AHRI)^11^ in uMkhanyakude in rural KwaZulu-Natal Province.

AHDSS has collected detailed, longitudinal data on vital events (births and deaths), migration and socioeconomic indicators since 1992. In 2017, the population was approximately 115 000 people.^12^ Updates occurred every 15–18 months from 1993 to 1998 and annually since 1999. About one-third of the population are Mozambique refugees who arrived in the early to mid-1980s following civil war, and their descendants. In the early to mid 1990s, the AHDSS population had longevity levels comparable to national estimates.^7^ From the late 1990s to mid-2000s, mortality increased in children and young and middle-aged adults due to the AIDS epidemic.^7 13^ ART became available in the site in three area hospitals in 2004 and in public clinics in 2008, becoming more widespread over time, with subsequent mortality declines.^7^ Compared with South Africa overall, the distribution of ages at death is broadly similar over time. Life expectancy at birth in AHDSS, however, has been consistently higher over time compared with another site in Mozambique (Manhiça site) and South Africa overall.^7 12^ The population has also experienced rapid socioeconomic development.^10^ A comparison of household asset indicators (eg, electricity and water availability) from AHDSS against tribal areas in Mpumalanga Province and South Africa showed that the prevalence of these indicators was also broadly comparable.^12^

AHRI began longitudinal monitoring of vital events, migration and socioeconomic indicators in 2000. Many socioeconomic indicators have significantly improved over time.^11^ For instance, access to electricity has increased to >95% and is broadly comparable to AHDSS and South Africa overall.^11 12^ In 2017, the population was approximately 145 000 people.^11^ Updates occurred twice yearly until 2012, and thrice yearly since then. In the early 2000s HIV/AIDS-related mortality was high in children and young adults (there was no population monitoring in the pre-HIV period).^8 13^ Public sector ART roll-out started in 2004 and mortality declined as ART became more widely available over time.^8^ Life expectancy at birth in AHRI has been consistently lower than AHDSS over time, although AHRI’s life expectancy began to increase in 2004 compared with 2008 for AHDSS.^12^ Similarly, child mortality in AHRI was higher than AHDSS until 2004.^14^ HIV prevalence among pregnant women in South Africa has also been among the highest in KwaZulu-Natal and Mpumalanga, exceeding 30% in 2013.^12^

In many resource-poor settings, VA interviews have been used to gather cause of death information in the absence of vital registration systems^15^ and have been widely applied by researchers in Health and Demographic Surveillance System sites.^16^ For deaths identified as occurring between census updates at both AHDSS and AHRI, a specially trained team or nurse conducted a VA with the closest living relative using a standardised VA instrument to record signs and symptoms experienced by the decedent prior to the death. The resulting VA data describe important signs and symptoms leading up to the death, which are then used in the assignment of cause of death described later.

### Data preparation

To interpret the VA data and assign cause of death, we used InSilicoVA,^17^ which uses a Bayesian model to estimate probabilities that the individual’s death was due to each of a list of possible causes (the probabilities sum to one over all causes). We generated separate estimates for each site, both of which included subpopulations of sex and age group (<1, ≥1–9 and ≥10 years). We aggregated the individual-level cause-specific probabilities by sex and age group and grouped causes of death into four broad categories aligned with the South African burden of disease classification^18^: HIV/AIDS/Tuberculosis (TB); other communicable diseases, maternal and perinatal conditions (excluding HIV/AIDS/TB; ICD); NCDs and external causes (EXT) (eg, accidents and injuries; EXT). We included HIV/AIDS and TB together because HIV is an underlying cause in many TB deaths and the VA method has difficulties distinguishing between HIV-related and non-HIV-related TB deaths. Therefore, this method yielded probabilities that a death in each sex/age group was due to each of the four cause categories.

We organised information on all individuals into person-years for each site, with one record for each full year lived and the year in which an individual died (including infants).

### Analytical approach

For five time periods that captured distinct periods of the pre and peak HIV/AIDS epidemic dynamics and changes due to ART availability,^7 8^ we calculated age-group-specific mortality rates (<1, 1–4, 5 years age groups 5–90, 95-plus) by site and sex, including: (1) 1994–1998 (pre-AIDS AHDSS); (2) 1999–2003 (emerging AIDS AHDSS; peak AIDS AHRI); (3) 2004–2008 (peak AIDS AHDSS; early ART AHRI); (4) 2009–2013 (early ART AHDSS; widespread ART AHRI) and (5) 2014–2018 (widespread ART AHDSS/AHRI). We fit a Kannisto model for ages 85–95+ to extend the upper age limit to 110+ per standard life table databases. We smoothed these mortality rates using cubic splines because sample sizes were small in some site-sex-age-period groups. We then restricted analysis to ages <85 given sparse data at older ages, possible bias due to age over-reporting and unreliable information on causes of death at oldest ages. For each site, sex and time period, we used standard methods to estimate abridged life tables and truncated life expectancy from birth to age 85 ( ${\;}_{85}e_{0}$ ). For each age group, the life table number of deaths ( ${\;}_{5}d_{a}$ ) and person-years lived in that age group ( ${\;}_{5}L_{a}$ ) are obtained, and LYL in that age group is calculated as $5{-}_{5}L_{a}$ .

We derived cause-specific mortality rates using the aggregated InSilicoVA model probabilities and followed the method proposed by Andersen *et al* 19 to calculate cause-specific LYL before age 85 ( ${\;}_{85}{ə}_{0}^{i}$ ). We distributed the life-table number of deaths in an age group using the proportion of observed deaths attributed to each cause and accumulated them into cause-specific LYL by age group and total LYL before age 85 (see online supplemental appendix for details). This method has the advantage of being additive: total LYL before age 85 plus truncated life expectancy sums to 85 ( ${\;}_{85}e_{0}+85{ə}_{0}=85$ ) and cause-specific LYL sums to total LYL ( ${\;}_{85}{ə}_{0}=\Sigma_{i=1}^{4}{\;}_{85}{ə}_{0}^{i}$ ). It also does not rely on the assumption of independence of competing risks for causes of death—where the removal of one cause leaves the risk of dying from all other causes unchanged. Using the life table age-at-death distribution avoids age structure confounding and facilitates comparisons between the sites over time. Since all terms used in calculating LYL are internal in the life table, LYL has a different interpretation than alternatives such as years of life lost^20^: as life expectancy corresponds to the average number of years lived in the population before age 85, similarly LYL corresponds to the complement, or average life-years lost in this population up to age 85. Our measure also captures both the magnitude and change in different causes of death over time, while approaches such as decomposing life expectancy by cause^8^ can only capture the contribution of changes in cause of death and requires that causes of death are independent.

10.1136/bmjgh-2023-013539.supp1 Supplementary data

We used SD of the age-at-death distribution as an indicator of mortality variation and modal age at death to measure location of the concentration of deaths. The selection of the mean, median and mode as a measure of central tendence to assess mortality changes depends on the purpose that the measure is to serve. For a description of the distribution of deaths, the mode is easily identified as the age with the highest value of the distribution. The mean, or life expectancy, is the expected value of the distribution, and predictions and inferences based on the mean have smaller SD than predictions based on the other values.^21^ Given these attributes, we focus our attention on the mean and the modal ages at death. We estimated 95% CIs for life expectancies and cause-specific LYL using 1000 Monte Carlo simulations. We used the statistical program R for all calculations.

### Patient and public involvement

Neither study participants nor public were involved in study design or conduct of the study. Both AHDSS and AHRI have ongoing liaison and open dialogue with their study communities and leaders.

## Results

**Figure 1:**
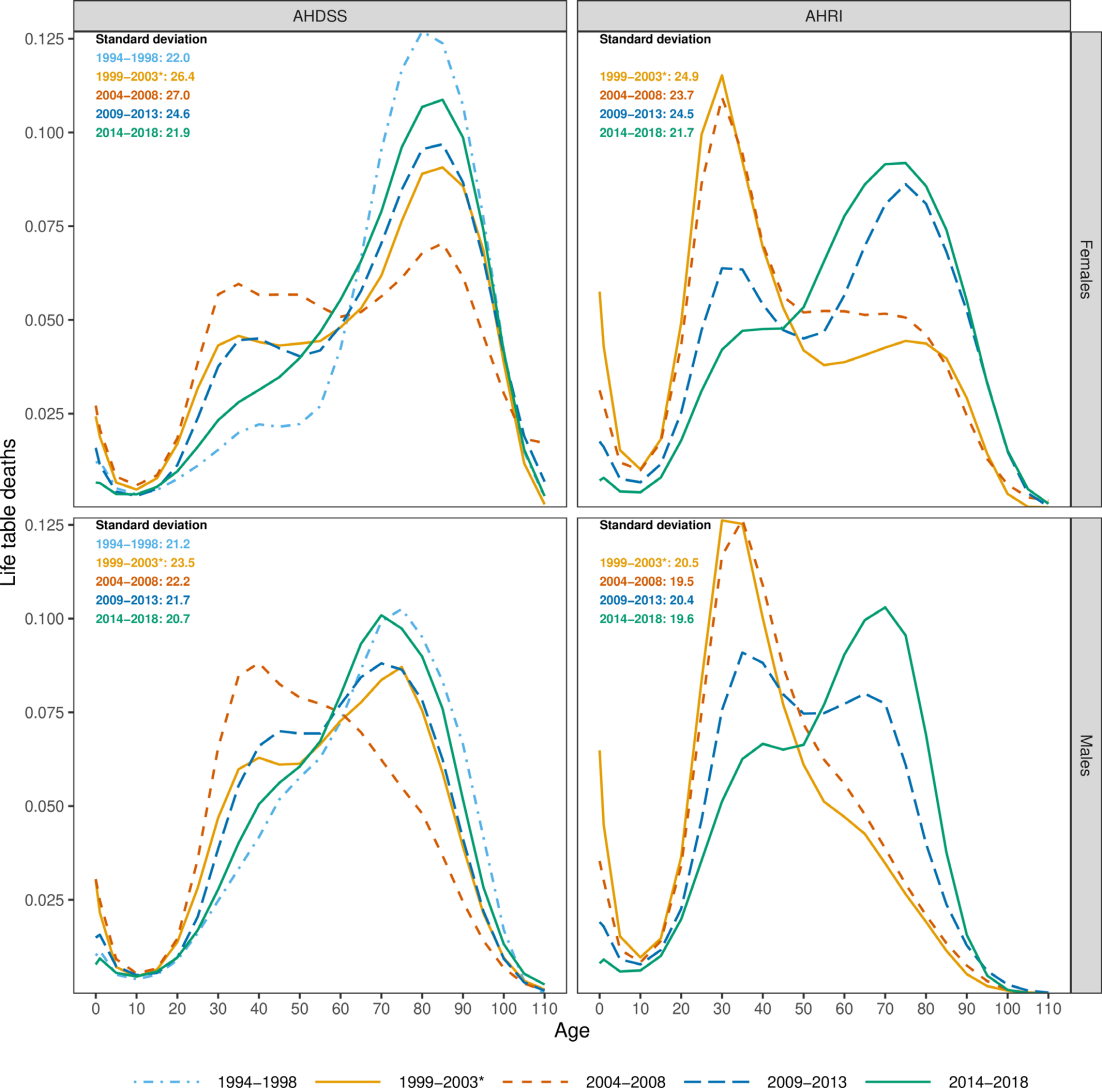
Life table age-at-death distributions and SD by sex and time period, Agincourt Health and Socio-Demographic Surveillance System (AHDSS) and Africa Health Research Institute (AHRI). *The time period 1999–2003 for AHRI only includes 2000–2003 as population surveillance commenced in 2000.

Figure 1 shows the life table age-at-death distribution and mortality variability by site, sex and time period. For AHDSS females, the peak of the AIDS epidemic (2004–2008) dramatically shifted the distribution of deaths to younger ages and increased mortality variability (SD). The modal age group at death was 80–84 in 1994–1998. The distribution changed to trimodal with peaks at ages 35–39 and 80–84 in 2004–2008 and high infant/child mortality. In 2014–2018, with widespread ART availability, the distribution and mode shifted back to older ages, but excess deaths in middle-ages remained. For males, the peak of the AIDS epidemic also shifted deaths to younger ages and increased mortality variability, but to slightly older ages compared with the shift for females. The modal age group at death shifted from 75 to 79 in 1994–1998 to 40–44 in 2004–2008. In the latest period, the distribution largely mirrored the period before the AIDS epidemic (1994–1998). Mortality variability in the latest period was similar to the pre-AIDS period for both females and males.

For AHRI, the AIDS epidemic (2000–2003) and early ART period (2004–2008) showed a modal age group at death of 30–34 for females. In the latest period the distribution of deaths shifted to older ages, with modal age 75–79. A similar pattern was evident for males, with death distribution peaks at 30–39 in the first two periods shifting to 70–74 in the widespread ART period. Mortality variability only started to decline from 2009 to 2013 onwards. For males and females in both sites, there was an excess proportion of deaths in the youngest ages during the AIDS periods without ART, which subsequently reduced in the latest ART period.

Table 1 shows that, for AHDSS, the AIDS epidemic substantially lowered truncated life expectancy from birth to age 85 ( ${\;}_{85}e_{0}$ ), with approximately 13-year declines for females and males compared with the pre-AIDS period and recovering to near pre-AIDS levels in the widespread ART period. For AHRI, ${\;}_{85}e_{0}$ increased during roll-out of ART time periods, though there was a persistent life expectancy gap compared with AHDSS in each time period (eg, in 2014–2018 a gap of about 6.7 years for females).

**Table 1:** Truncated life expectancy ( ${\;}_{85}e_{0}$ ) and life-years lost ( ${\;}_{85}{ə}_{0}$ ) trends (ages 0–85) by sex and time period, Agincourt Health and Socio-Demographic Surveillance System (AHDSS) and Africa Health Research Institute (AHRI)

	AHDSS Estimate (95% CI)					AHRI Estimate (95% CI)			
	1994–1998	1999–2003*	2004–2008	2009–2013	2014–2018	2000–2003*	2004–2008	2009–2013	2014–2018
Females									
Life expectancy	71.4 (70.6 to 72.3)	62.9 (62.0 to 63.8)	58.9 (58.0 to 59.7)	65.8 (65.1 to 66.6)	69.3 (68.7 to 70.0)	44.3 (43.5 to 45.2)	47.9 (47.2 to 48.7)	58.5 (57.7 to 59.3)	62.6 (61.9 to 63.3)
Life-years lost	13.6 (12.7 to 14.4)	22.1 (21.2 to 23.0)	26.1 (25.3 to 27.0)	19.2 (18.4 to 19.9)	15.7 (15.0 to 16.3)	40.7 (39.8 to 41.5)	37.1 (36.3 to 37.8)	26.5 (25.7 to 27.3)	22.4 (21.7 to 23.1)
HIV/AIDS/TB	4.1 (3.8 to 4.5)	11.0 (10.5 to 11.6)	13.9 (13.4 to 14.5)	6.4 (6.1 to 6.7)	3.2 (3.0 to 3.4)	23.8 (23.1 to 24.5)	20.2 (19.6 to 20.8)	12.7 (12.2 to 13.3)	8.9 (8.5 to 9.4)
ICD	3.4 (3.1 to 3.7)	4.4 (4.1 to 4.8)	5.8 (5.4 to 6.2)	5.5 (5.1 to 5.8)	4.5 (4.3 to 4.8)	8.6 (8.0 to 9.3)	6.5 (6.0 to 6.9)	4.3 (4.0 to 4.7)	2.8 (2.6 to 3.1)
NCD	4.9 (4.5 to 5.2)	5.5 (5.2 to 5.8)	5.2 (4.9 to 5.4)	6.2 (5.9 to 6.4)	6.8 (6.5 to 7.1)	6.2 (6.0 to 6.5)	8.5 (8.2 to 8.8)	7.5 (7.2 to 7.8)	8.9 (8.5 to 9.2)
EXT	1.1 (1.0 to 1.2)	1.1 (1.0 to 1.2)	1.2 (1.1 to 1.3)	1.1 (1.0 to 1.2)	1.1 (1.1 to 1.2)	2.0 (1.8 to 2.1)	1.9 (1.7 to 2.0)	2.0 (1.8 to 2.1)	1.8 (1.6 to 1.9)
Males									
Life expectancy	65.1 (64.2 to 66.2)	57.9 (57.0 to 58.8)	52.6 (51.9 to 53.4)	60.0 (59.3 to 60.7)	63.8 (63.0 to 64.5)	40.2 (39.5 to 40.9)	43.7 (43.1 to 44.4)	51.6 (50.8 to 52.3)	57.6 (56.8 to 58.3)
Life-years lost	19.9 (18.8 to 20.8)	27.1 (26.2 to 28.0)	32.4 (31.6 to 33.1)	25.0 (24.3 to 25.7)	21.2 (20.5 to 22.0)	44.8 (44.1 to 45.5)	41.3 (40.6 to 41.9)	33.4 (32.7 to 34.2)	27.4 (26.7 to 28.2)
HIV/AIDS/TB	5.8 (5.5 to 6.2)	11.0 (10.5 to 11.4)	14.5 (14.0 to 15.0)	7.0 (6.7 to 7.3)	4.1 (3.8 to 4.3)	21.9 (21.3 to 22.5)	19.1 (18.5 to 19.6)	15.1 (14.6 to 15.6)	9.8 (9.4 to 10.2)
ICD	3.3 (3.0 to 3.6)	5.5 (5.1 to 6.0)	6.9 (6.5 to 7.3)	5.9 (5.6 to 6.3)	5.2 (4.9 to 5.5)	8.7 (8.0 to 9.4)	6.2 (5.7 to 6.6)	4.1 (3.8 to 4.5)	2.8 (2.5 to 3.0)
NCD	6.8 (6.4 to 7.1)	6.7 (6.4 to 7.0)	7.2 (6.9 to 7.5)	8.7 (8.4 to 9.0)	8.5 (8.2 to 8.8)	7.2 (6.9 to 7.5)	9.0 (8.6 to 9.3)	7.7 (7.5 to 8.0)	7.9 (7.6 to 8.2)
EXT	4.0 (3.7 to 4.3)	3.8 (3.6 to 4.1)	3.7 (3.5 to 4.0)	3.4 (3.2 to 3.7)	3.5 (3.3 to 3.7)	6.9 (6.6 to 7.4)	7.1 (6.7 to 7.5)	6.4 (6.1 to 6.8)	6.9 (6.6 to 7.3)

*The first time period for AHRI only includes 2000–2003 as population surveillance commenced in 2000.

EXT, external causes; ICD, other communicable diseases, maternal and perinatal conditions; NCD, non-communicable diseases.

**Figure 2:**
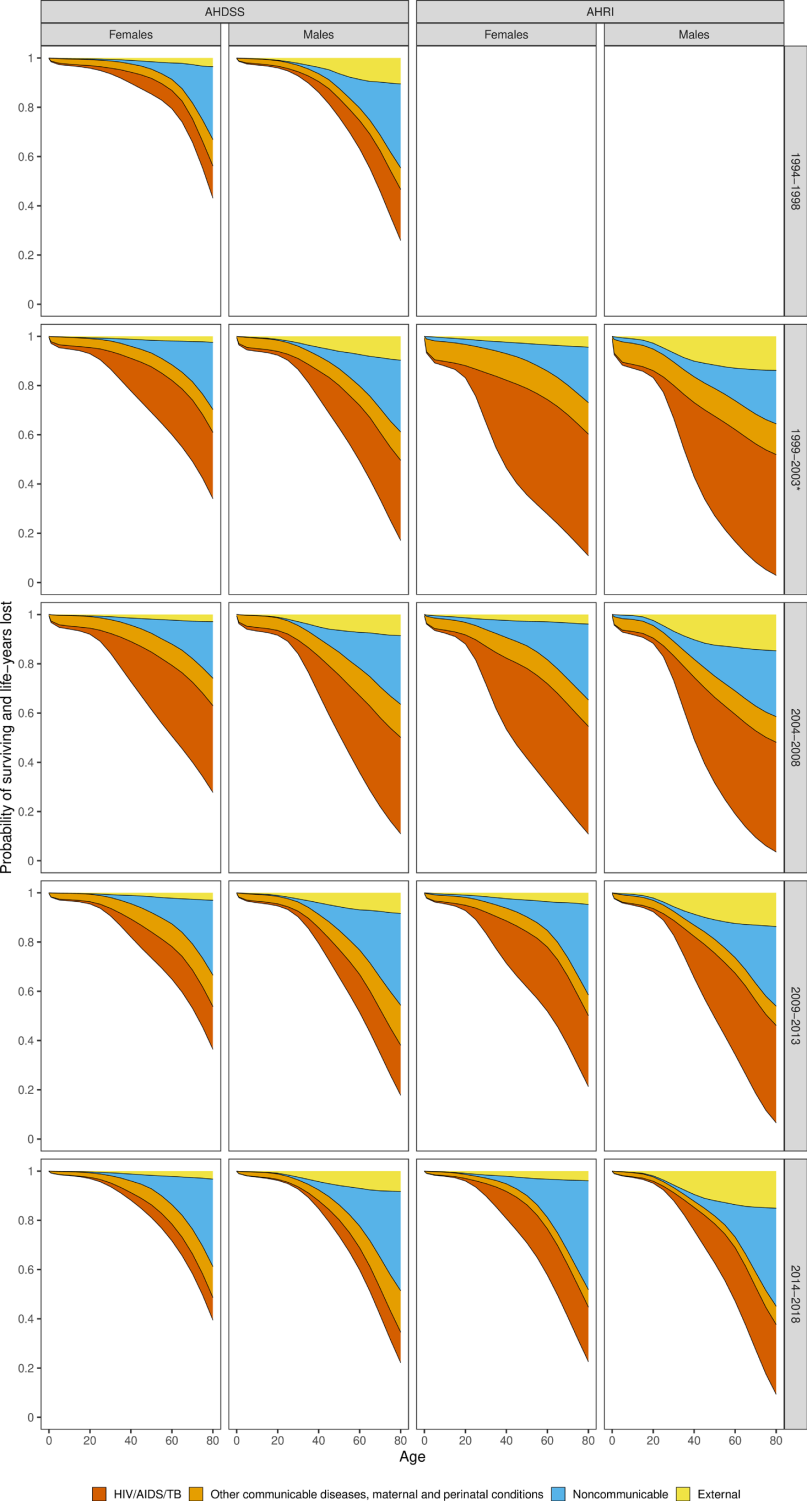
Probabilities of survival and deaths from different causes for individuals aged 0–85 years by sex and time period, Agincourt Health and Socio-Demographic Surveillance System (AHDSS) and Africa Health Research Institute (AHRI). *The time period 1999–2003 for AHRI only includes 2000–2003 as population surveillance commenced in 2000. TB, Tuberculosis.

Figure 2 presents the life table survival function, with ${\;}_{85}e_{0}$ as the area below the lowest curve and total LYL the area above the curve. Figure 2 and table 1 show that for both sites the total LYL due to HIV/AIDS/TB was similar for both sexes (eg, 2004–2008 AHDSS 13.9 years (95% CI 13.4 to 14.5) for females, 14.5 (95% CI 14.0 to 15.0) males; AHRI 20.2 (95% CI 19.6 to 20.8) females, 19.1 (95% CI 18.5 to 19.6) males). At AHDSS, in the latest time period, LYL due to NCDs exceeded HIV/AIDS/TB for both females and males for the first time since the epidemic started. At AHRI, even in the latest time period LYL due to HIV/AIDS/TB continued to exceed all other causes for males and was equal to LYL due to NCDs for females.

**Figure 3:**
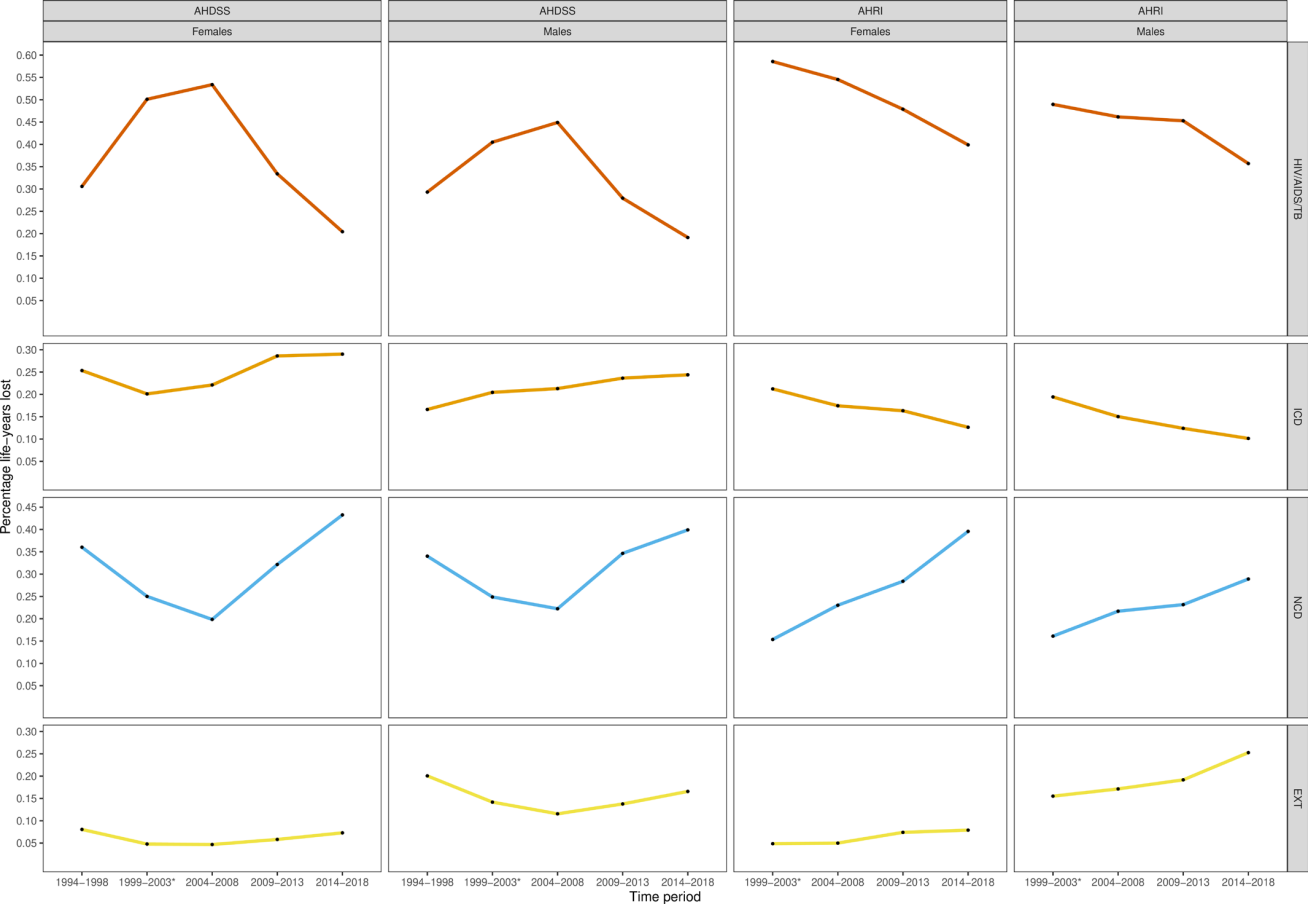
Percentage of life-years lost by cause of death by sex and time period, Agincourt Health and Socio-Demographic Surveillance System (AHDSS) and Africa Health Research Institute (AHRI). *The time period 1999–2003 for AHRI only includes 2000–2003 as population surveillance commenced in 2000. TB, Tuberculosis; EXT, external causes; ICD, other communicable diseases, maternal and perinatal conditions; NCD, non-communicable diseases.

Figure 3 shows the proportion of LYL attributed to each cause by site, sex and time period. For AHDSS, HIV/AIDS/TB deaths accounted for an increasing share of LYL until the peak of the AIDS epidemic, with substantial declines in the early and widespread ART periods. The share of LYL due to other communicable diseases increased until the period of widespread ART. For females, a greater proportion of LYL was due to HIV/AIDS/TB compared with males (53% vs 45% in 2004–2008). For both sexes, the share of LYL due to NCDs declined alongside the AIDS epidemic rise and peak, and then exceeded the shares in the pre-AIDS period during the era of widespread ART (43% for females and 40% for males). For AHRI we lack a pre-AIDS time period. During 2000–2003, HIV/AIDS/TB and other communicable diseases were the principal drivers of LYL. The share of LYL due to HIV/AIDS/TB and other communicable diseases declined alongside the ART roll-out periods, though females continued to have a greater share of their LYL due to HIV/AIDS/TB compared with males. For females, the share from HIV/AIDS/TB declined from 59% in 2000–2003 to 40% in 2014–2018. For males, the share from HIV/AIDS/TB declined from 49% to 36% over the same periods. EXT such as homicide contributed a substantial proportion of LYL for males at both sites, accounting for an increasing share in the most recent time periods (17% AHDSS; 25% AHRI).

**Figure 4:**
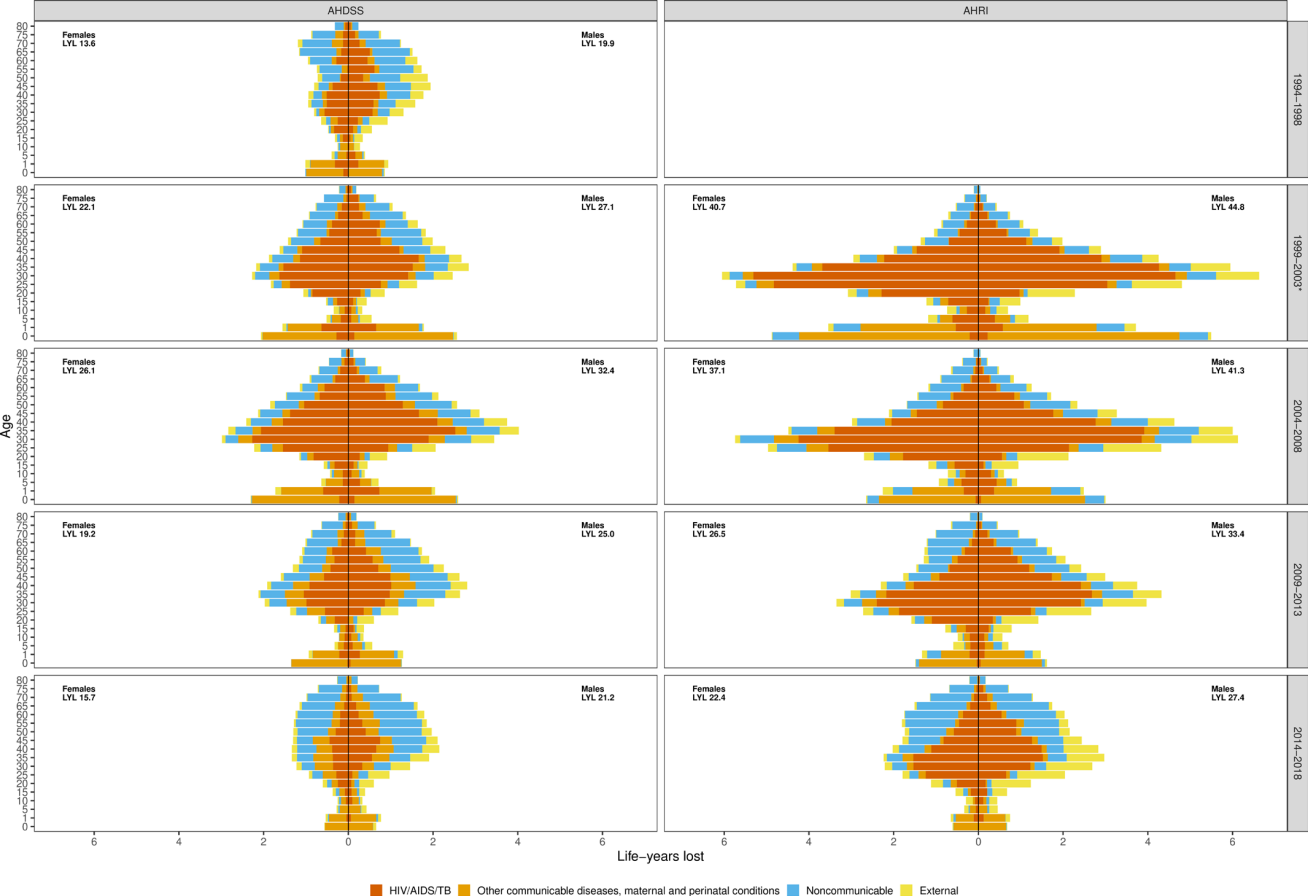
Contribution of life-years lost by time period, sex, age at death and cause of death at Agincourt Health and Socio-Demographic Surveillance System (AHDSS) and Africa Health Research Institute (AHRI). *The time period 1999–2003 for AHRI only includes 2000–2003 as population surveillance commenced in 2000. LYL in an age group were distributed to the four causes using the proportion of deaths attributed to each cause (see appendix for calculation details). TB, Tuberculosis; LYL, life-years lost.

Figure 4 shows the contribution of cause-specific LYL by age group. At AHDSS, for females in the pre-AIDS period children under 5 contributed the most LYL (15%) until ages 65–75. For males, while LYL under age 5 was an important contributor, ages 30–70 contributed greater shares of LYL due to HIV/AIDS/TB, EXT and NCDs in older ages. The early and peak AIDS periods showed high shares of LYL for children, primarily due not to HIV/AIDS but to other communicable diseases, and middle-aged adults due to HIV/AIDS/TB. At AHRI, about 20% of LYL were contributed by young children in 2000–2003, largely due to other communicable diseases, which declined in each subsequent period. The very high LYL during 2000–2003 and 2004–2008 was largely concentrated in middle-aged adults. Both females and males lost about 50% of their life-years between ages 25 and 45 in these periods. EXT represented an important share of LYL for young and middle-aged men in all periods. For both sites, LYL due to HIV/AIDS/TB was concentrated at earlier ages for females compared with males, for example, AHRI females lost 10.1 years due to HIV/AIDS/TB at ages 25–35 in 2000–2003 compared with about 7.7 years for males.

## Discussion

Our comparative results highlight dramatic changes in life expectancy and mortality variability by age and cause of death over a quarter century in rural South Africa. The AIDS epidemic shifted the mortality distribution towards younger ages at both sites, resulting in greater variability in age-at-death. In subsequent time periods of widespread availability of ART, life expectancy substantially increased and LYL declined, primarily through reductions in LYL due to HIV/AIDS/TB in middle ages and other communicable diseases in young children.

Comparisons between sites showed large differences in the changing burden of LYL over time. The greater LYL due to HIV/AIDS/TB at AHRI compared with AHDSS reflects in part historical differences and changes in HIV infection and prevalence.^22^ Comparative evidence from 2010 to 2011 showed a markedly different sex and age pattern of HIV prevalence, where prevalence was concentrated in younger ages at AHRI while AHDSS had concentrations in middle and older ages.^23^ While the ART periods were associated with substantial reductions in LYL due to HIV/AIDS/TB at both sites, AHDSS experienced greater decline. ART roll-out occurred earlier at AHRI, so these differences likely reflect differences in other factors such as ART eligibility thresholds, availability of treatment regimens, and improvements in engagement with HIV treatment services.^8^ At AHRI the burden of LYL due to HIV/AIDS/TB persisted, which may be due in part to HIV/AIDS/TB mortality increasingly being concentrated in those who have not started treatment.^24^ Evidence from the ALPHA Network, however, indicates that pretreatment mortality among those living with HIV is declining rapidly.^25^ Persistent disparities in HIV/AIDS/TB mortality highlight the importance of continuing to monitor progress in successfully transitioning individuals through the HIV care cascade and attaining the 90-90-90 UNAIDS goals.

Even in the latest time period available for this analysis, important age differentials in LYL persist at each site. At AHDSS, these differentials are largely driven by LYL due to NCDs. As survival gains continue to increase for people living with HIV and with the subsequent ageing of this population, greater attention is needed to disentangle any potential interaction of HIV, ART and NCDs in older ages.^26^ This is even more salient given the ongoing COVID-19 pandemic that began after the period covered in this study. A South African study of in-hospital mortality rates among patients with COVID-19 found increased mortality risk for people with HIV (particularly for those not on ART) and for those with chronic comorbidities.^27^ Evidence from AHDSS indicates a high level of multimorbidity and high prevalence of HIV in older adults.^28^ A population-based study at AHRI also found a high burden of multimorbidity, with those living with HIV and on ART generally well controlled but a majority of those with conditions such as hypertension not optimally controlled.^29^ In order to further reduce mortality disparities as LYL continues to concentrate in NCDs, synergies in bundling treatments together can help reduce the burden of managing multiple chronic conditions.

At the peak of the AIDS epidemic the majority of LYL in young children was due to other communicable diseases (though under-reporting of HIV/AIDS-related deaths may have occurred). This could partly reflect elevated mortality risk due to either a terminally ill or deceased caregiver.^13^ Earlier work at AHRI showed significant declines in infant mortality after ART roll-out due to treatment of HIV-infected mothers.^30^ In relation to the COVID-19 pandemic, our finding raises the importance of further investigating the immediate and longer-term impacts of caregiver COVID-19-associated deaths on children—particularly as results from a modelling study also suggest a high burden of these deaths in South Africa.^31^ While LYL in young children declined at each site since ART roll-out,^13^ our findings highlight the unfinished agenda of improving child mortality. Moreover, comparisons between AHDSS and national data,^7^ and a study comparing antenatal care registers with DSS data from Kenya,^32^ suggest that our estimates of LYL for children are conservative given likely downward bias in DSS estimates of mortality from missing pregnancy reports and newborn deaths.

Shares of LYL due to EXT increased in the latest time periods, particularly for males. The burden of violence and injuries in South Africa is high and disproportionately focused in young men, as both victims and perpetrators,^33^ becoming an increasingly important share of mortality variability, particularly at AHRI. Further research is needed to identify the principal causes of external deaths (homicides, suicides and accidents) in these sites over time. Furthermore, while mortality is the most severe outcome of violence, it underestimates the full toll among survivors who suffer from disability and mental trauma, among other outcomes.^5^ Our results highlight the importance of understanding the underlying determinants of premature mortality due to EXT and its impact on the younger population.

Our results complement and extend prior evidence on the role of ART in AIDS-related mortality at AHRI. Reniers *et al* 8 used AHRI data through 2014 to decompose life expectancy changes by cause of death and compare the overall and HIV-negative populations. We extend their approach by quantifying the burden of mortality due to different causes of death at different periods of time and the changes in the burden over time. We also complement these analyses by showing how the age profile of mortality changed over time. Finally, our results provide an update to mortality changes at AHRI since 2014 and a comparison with AHDSS, which provides an important mortality baseline as it includes a time period before the AIDS epidemic.

Our findings also provide a fundamental baseline for studies examining how the COVID-19 pandemic has changed the burden and distribution of deaths and resulting mortality variation. This is particularly important in comparing intraregional and subnational mortality patterns, given varying demographic, socioeconomic and morbidity population profiles, government suppression and mitigation strategies, and healthcare treatment and systems reorientation. Despite data limitations in much of sub-Saharan Africa, excess mortality estimates for South Africa in 2020–2021^34^ suggest that the pandemic had a substantial impact and our results provide an important comparison in interpreting evolving mortality differentials.

Reductions in HIV/AIDS/TB mortality in later periods, while likely due to ART availability, may also reflect improvements in TB treatment programmes. People living with HIV may benefit indirectly from engaging with healthcare services. Evidence from an AHDSS cohort study of older adults suggests people living with HIV and engaged with care are more likely to be aware of a hypertension diagnosis and engaged in hypertension treatment,^35^ which is particularly salient given the high prevalence and incidence of hypertension in this population.^28^

As people living with HIV survive to older ages, attribution of causes of death becomes more complicated, given comorbidities. Our approach to changes in causes of death is inherently limited by use of VAs. By using broad cause of death categories and focusing on LYL before age 85, we have mitigated the misclassification of causes, with most of the reduction in LYL attributed to HIV/AIDS/TB and other communicable diseases. Our results may not agree with studies that do not measure mortality variation using SD and LYL. However, studies have indicated broad agreement between other measures of mortality variability,^3^ and our LYL measure closely aligns with life disparity as an indicator of mortality variation.^19^ Finally, we were unable to include HIV status in our mortality comparisons as routine HIV testing during census rounds is not done at AHDSS. This restricted the possibility of within population comparisons of individuals with and without HIV.^8 36^

As the South African epidemiological and health transition continues to evolve—with the world’s largest ART programme and concomitant survival gains and the ageing of the HIV epidemic^22^—continued monitoring of mortality variability is critical to understand persistent and emerging differentials in survival.^3^ Our finding of evolving yet persistent burdens in mortality differentials at different ages, alongside emerging evidence of changes in the demographics of the HIV epidemic,^22^ suggests greater prevention efforts are needed at middle and older ages and greater mitigation of disease in children. Integrated and targeted public health programmes are needed as infectious and NCD epidemics increasingly overlap in South Africa.^29^

## Data Availability

Data are available in a public, open access repository. Aggregated data are available from the Africa Health Research Institute (AHRI) Data Repository (data.ahri.org): doi: https://doi.org/10.23664/AHRI.RD01-01.agin-aggregate-01.%5B9%5D Individual-level data for AHRI are available from the AHRI Data Repository for researchers who meet the criteria for access to confidential data and sign on the agreement according to AHRI’s policy for data sharing. Customised data extraction can be requested from AHRI data management service desk (rdmservicedesk@ahri.org). Detailed documentation of the AHDSS data and an anonymised database containing data from 10% of the surveillance households are available for public access on the AHDSS website (http://www.agincourt.za). The AHDSS core demographic data are also routinely deposited for public access in the INDEPTH Network Data Repository (http://www.indepth-ishare.org/) and the SAPRIN Data Repository (http://saprindata.samrc.ac.za/index.php/catalog). Customised data extraction can be requested from FXG-O (F.Gomez-OliveCasas@wits.ac.za).
