# Supplementary material for: Mortality variability and differentials by age and causes of death in rural South Africa, 1994–2018
Source: BMJ Glob Health. 2024 Apr 8;9(4):e013539. doi: 10.1136/bmjgh-2023-013539 (PMC11015189; doi:10.1136/bmjgh-2023-013539)
Supplement: Supplementary data [file bmjgh-2023-013539supp001.pdf]

Mortality variability and differentials by age and causes of death in rural South Africa, 1994 – 2018 – appendix

Brian Houle,[1,2,3] PhD
Chodziwadziwa W Kabudula,[2] PhD
Sanyu A Mojola,[4] PhD
Nicole Angotti,[5,2,3] PhD
F Xavier Gómez-Olivé,[2] PhD
Dickman Gareta,[6] MSc
Kobus Herbst,[6,7] MSc
Samuel J Clark,[8,2] PhD
Jane Menken,[3,2] PhD
Vladimir Canudas-Romo,[1] PhD

1 School of Demography, The Australian National University, Canberra, Australia
2 MRC/Wits Rural Public Health and Health Transitions Research Unit (Agincourt), Faculty of Health Sciences, School of Public Health, University of the Witwatersrand, Johannesburg, South Africa
3 Institute of Behavioral Science, University of Colorado Boulder, Boulder, Colorado, United States
4 Department of Sociology, School of Public and International Affairs, and Office of Population Research, Princeton University, Princeton, New Jersey, United States
5 Department of Sociology, American University, Washington, D.C., United States
6 Africa Health Research Institute, Durban, South Africa
7 DSI-MRC South African Population Research Infrastructure Network, Durban, South Africa
8 Department of Sociology, The Ohio State University, Columbus, Ohio, United States

1 Life tables and truncated metrics 2
2 Age- and cause-specific life-years lost (LYL) 3
3 Confidence intervals (CI) 5
4 Comparison with InterVA-5 6
5 Cause of death classification 10

Houle B, *et al. BMJ Glob Health* 2024; 9:e013539. doi: 10.1136/bmjgh-2023-013539

1 Life tables and truncated metrics

We estimated life tables using standard methods and truncated life expectancy from birth to age 85, denoted ${}_{85}\mathrm{e}_0 = \int_0^{85} \ell(x)\,dx$, where $\ell(x)$ is the life table survival function and the radix of the population is zero, $\ell(0) = 1$. Additionally, the life table distribution of deaths, or $f(x) = \ell(x) - \ell(x+1)$, was used to calculated the modal age at death or age where the maximum $f(x)$ is found, and the standard deviation from age 0 to 85, as $SD = \sqrt{\int_0^{85} (x - {}_{85}\mathrm{e}_0)^2 f(x)\,dx}$.

Houle B, *et al. BMJ Glob Health* 2024; 9:e013539. doi: 10.1136/bmjgh-2023-013539

2 Age- and cause-specific life-years lost (LYL)

We used cause-specific life-years lost before age 85, or ${}_{85}\text{ə}_0^i$, to assess the contribution of each cause of death to the total life span variation.[1] We calculated the life-years lost due to different causes of death by focusing on the cumulative life table distribution of deaths, or $f(0,x) = \int_0^x f(a)\, da$ from age 0 to x, which was further subdivided into the different causes of death, or $f(0,x,i)$ for cause i. For our 4 mutually exclusive causes of death, the number of life-years lost to each cause of death between ages 0 and 85 was calculated as ${}_{85}\text{ə}_0^i = \int_0^{85} f(0,x,i)dx$. When added over all causes, the overall life-years lost is the complement of the truncated life expectancy ${}_{85}e_0$ between these ages, as they add to 85 years, ${}_{85}\text{ə}_0 = \sum_{i=1}^4 {}_{85}\text{ə}_0^i$. Additionally, we also studied the age-contribution of life-years lost as the product of the age- and cause-specific life table distribution of deaths, or $f(x,i)$ at age x and cause i, with the remaining years to age 85, or (85-x). In the latter perspective, the life-years lost to each cause of death between ages 0 and 85 are calculated as ${}_{85}\text{ə}_0^i = \int_0^{85} f(x,i)(85-x)dx$.

Figures A1 and A2 present the two perspectives of looking at life-years lost: horizontally slicing - using the life table distribution of deaths, $f(x)$; or vertically slicing – using its cumulative life table distribution of deaths $f(0,x)$. As observed in Figure A1 the two perspectives are equivalent and return the same total life-years lost, ${}_{85}\text{ə}_0$. However, the age-contribution allows us to additionally quantify the lifespan burden of life-years lost by age.

Figure A1. Horizontally slicing calculations of life years lost.

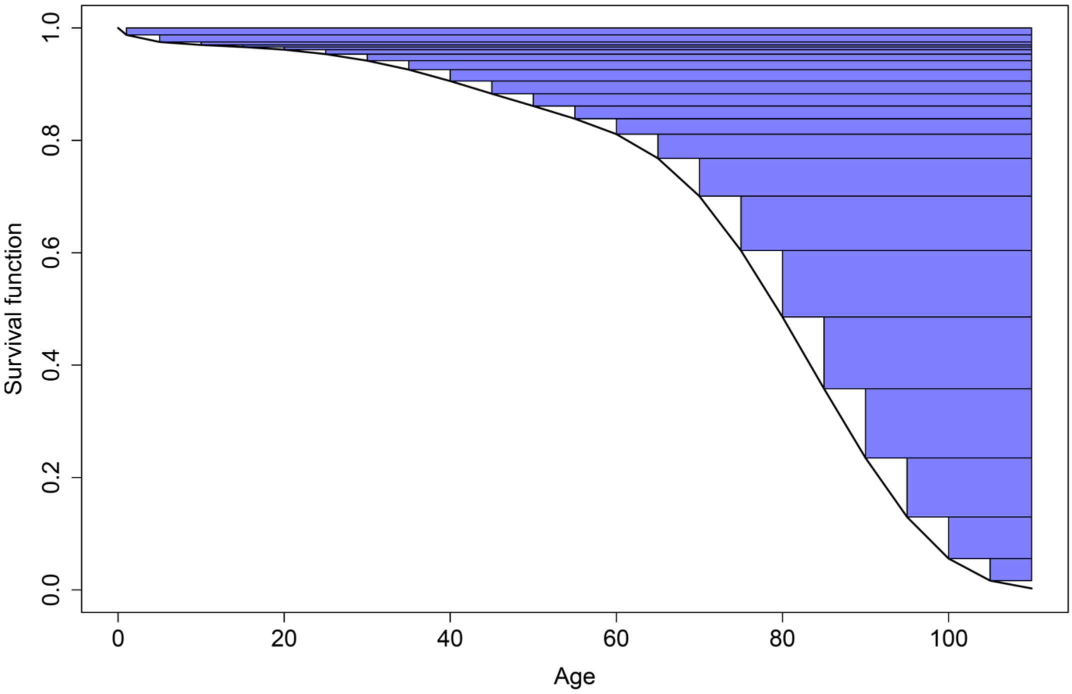

Figure A2. Vertically slicing calculations of life-years lost.

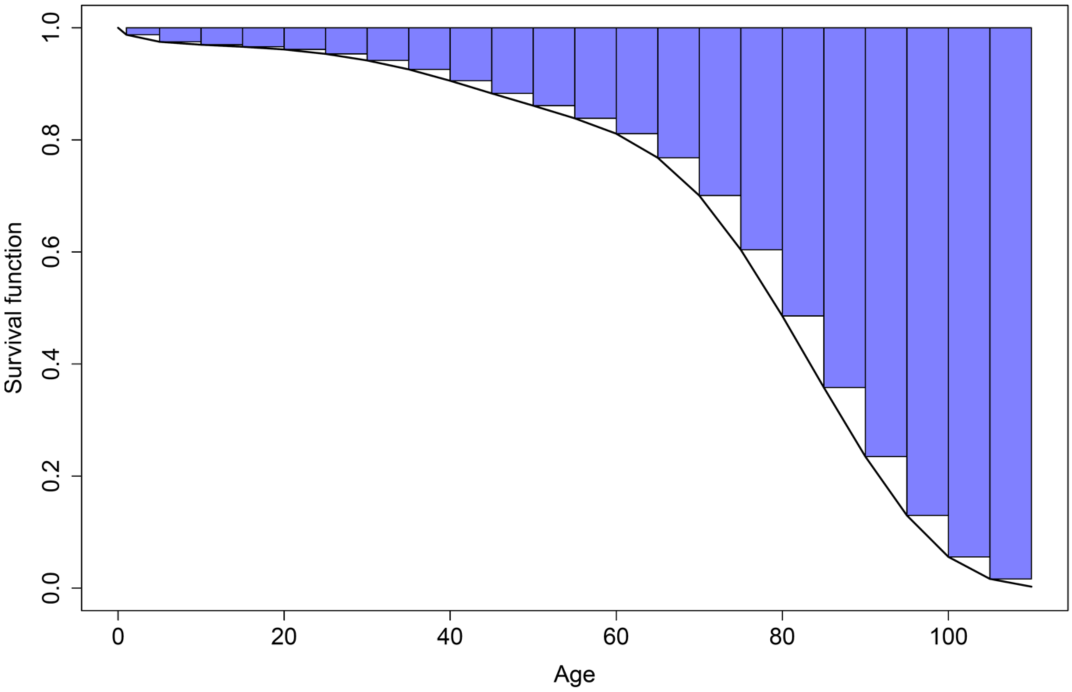

Formally, this can be shown by noticing the analogy of life expectancy which can be calculated as the average years lived based on the survival function (vertically slicing) or as the average age at death based on the life table distribution of deaths (horizontally slicing)

$$_{85}\mathrm{e}_0 = \int_0^{85} \ell(x)\, dx = \int_0^{85} x f(x) dx.$$

Similarly, life-years-lost can be calculated as the complement of the survival function (vertically slicing) or as the complement to age 85 from the age at death distribution (horizontally slicing), as

$$_{85}\mathrm{ə}_0 = \int_0^{85} [1 - \ell(x)]\, dx = \int_0^{85} f(0, x)\, dx = \int_0^{85} f(x)(85 - x) dx.$$

Houle B, *et al. BMJ Glob Health* 2024; 9:e013539. doi: 10.1136/bmjgh-2023-013539

3 Confidence intervals (CI)

The uncertainty in life table measures was estimated with a simulation approach by generating 1000 draws from the distribution of sex, site, and time-period age-specific deaths and death rates for each population using the binomial distribution. With each draw of the death rates, a simulated life table was created. We derived 95% uncertainty intervals from the 2.5 and 97.5 percentiles of the resulting 1,000 estimates of life expectancy and the other life table metrics.[2] To compute the uncertainty of life-years lost by causes of death, we further resampled death numbers for each cause of death (1,000 iterations) using multinomial distributions, conditional on sex, site, time-period and age-groups.[3]

4 Comparison with InterVA-5

Figures A3-A5 show the cause of death figures presented in the manuscript using InterVA-5[4] as an alternative model for cause of death attribution. This allows for an assessment of the sensitivity of the results to the choice of VA model, as well as comparison with earlier work that has used InterVA instead of InSilicoVA. For each death with a complete verbal autopsy interview, the model assigned up to three likely causes of death consistent with the signs and symptoms or an indeterminate cause of death. InterVA-5 was run with malaria prevalence set to low and HIV prevalence set to high. We then grouped causes of death into the following broad categories using the same approach as in the manuscript with the addition of an indeterminate group: HIV/AIDS/TB; other communicable diseases, maternal and perinatal conditions (excluding HIV/AIDS/TB; ICD); noncommunicable diseases (NCD); external causes (e.g., accidents and injuries; EXT); and indeterminate causes (IND). Overall, the results are similar to the results from InSilicoVA presented in the manuscript. The main difference is the classification of deaths as indeterminate in the InterVA approach, particularly in older ages.

Figure A3. Probabilities of survival and deaths from different causes for individuals aged 0-85 years by sex and time-period based on InterVA-5 results, Agincourt Health and socio-Demographic Surveillance System (AHDSS) and Africa Health Research Institute (AHRI).

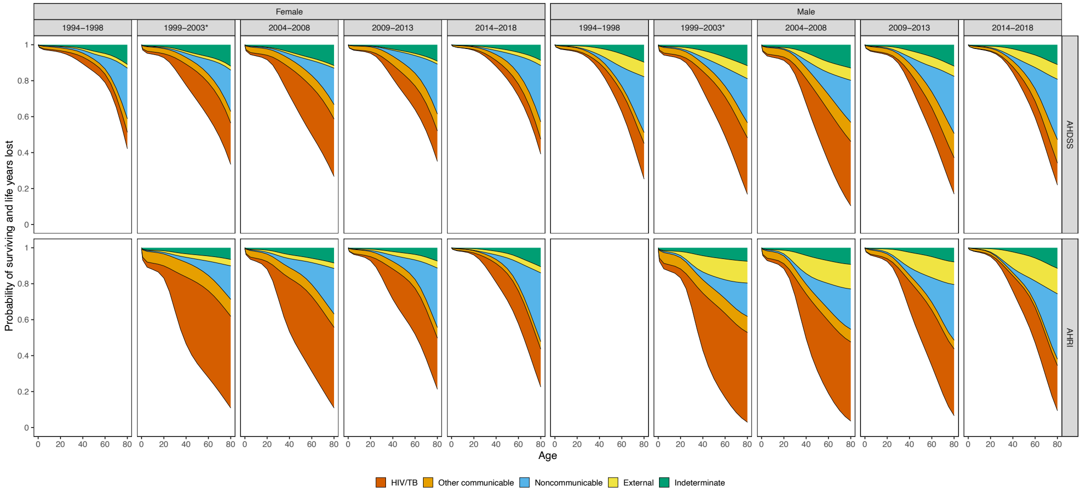

* The time-period 1999-2003 for AHRI only includes 2000-2003 as population surveillance commenced in 2000.

Houle B, *et al. BMJ Glob Health* 2024; 9:e013539. doi: 10.1136/bmjgh-2023-013539

Figure A4. Percentage of life-years lost by cause of death by sex over time at Agincourt Health and socio-Demographic Surveillance System (AHDSS) and Africa Health Research Institute (AHRI) based on InterVA-5 results.

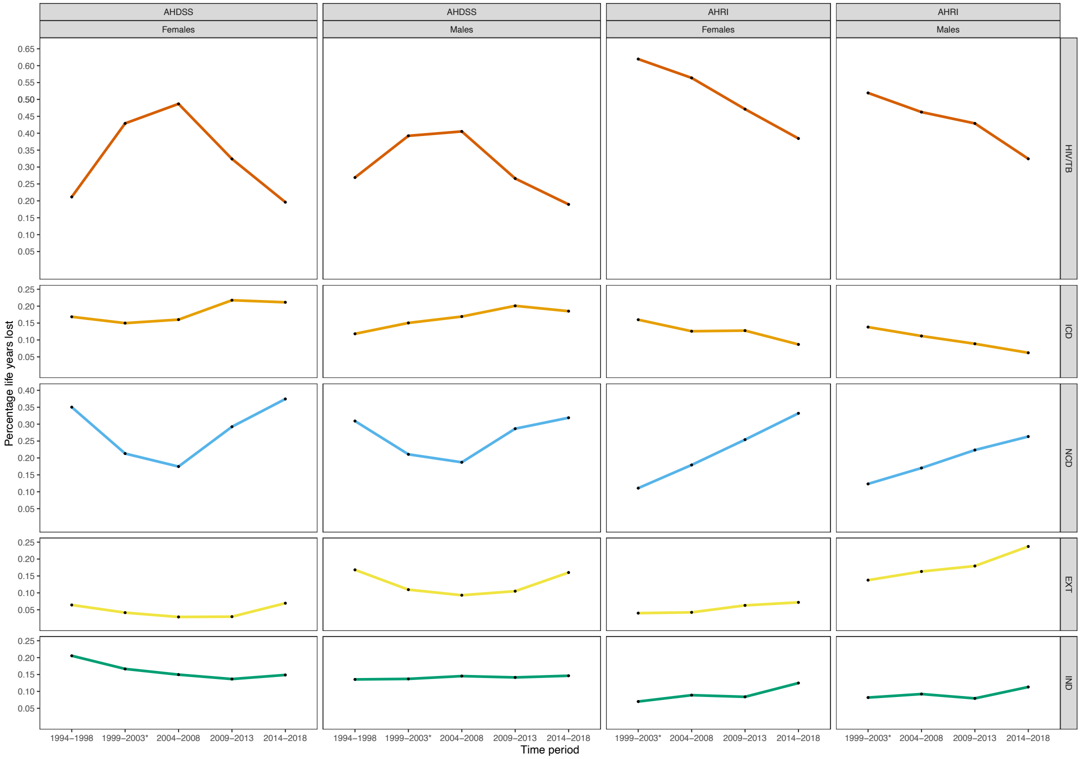

* The time-period 1999-2003 for AHRI only includes 2000-2003 as population surveillance commenced in 2000. ICD: other communicable diseases, maternal and perinatal conditions; NCD: noncommunicable diseases; EXT: external causes; IND: indeterminate.

Figure A5. Contribution of life-years lost by age group, sex, time-period, and cause of death at Agincourt Health and socio-Demographic Surveillance System (AHDSS) and Africa Health Research Institute (AHRI) based on InterVA-5 results.

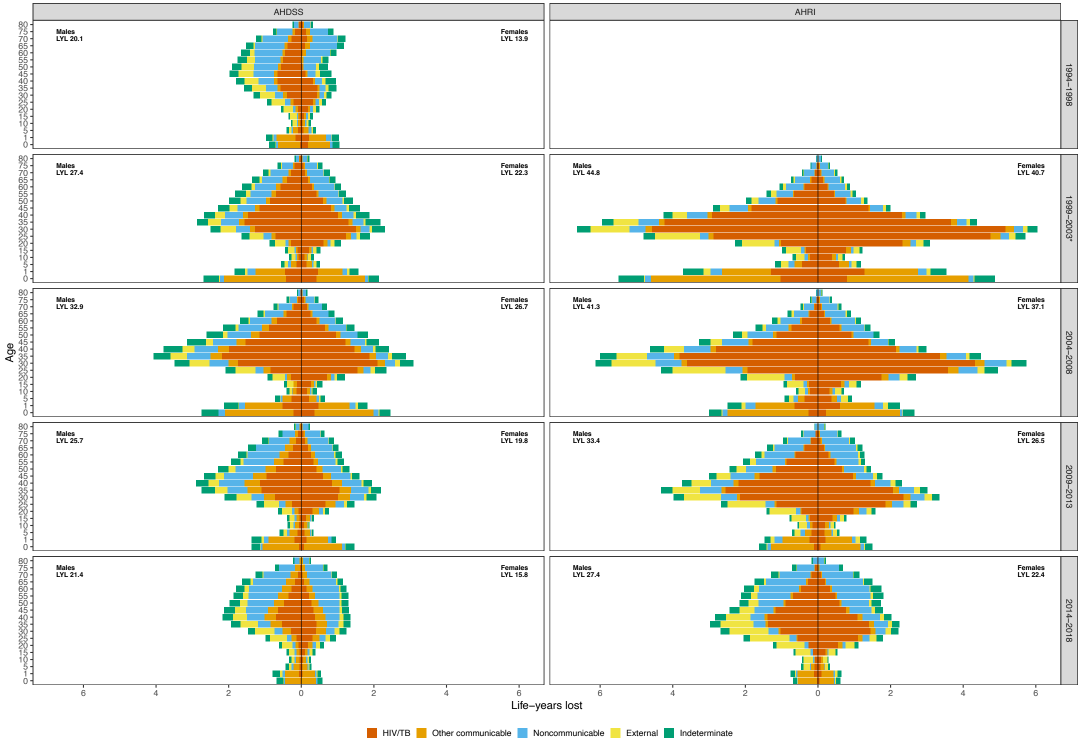

* The time-period 1999-2003 for AHRI only includes 2000-2003 as population surveillance commenced in 2000.

Appendix, p9

5 Cause of death classification

Table A1 shows how the cause of death groups correspond to their ICD-10 codes.

Table A1. Cause of death classification

COD groups	ICD-10 codes
HIV/AIDS/TB	A15-A16; B20-B24
Other communicable diseases, maternal and perinatal conditions (ICD)	A00-A09; A17-A99; B00-B19; B25-B99; D50-D64; E40-E46; G00-G05; J00-J22; O00-O08; O10-O16; O20-O99; P00-P15; P20-P96; Q00-Q99
Noncommunicable diseases (NCD)	C00-C26; C30-C58; C60-D48; D55-D89; E00-E07; E10-E35; E50-E90; F00-F99; G06-G37; G40-G41; G50-G99; H00-H95; I00-I15; I20-I52; I60-I99; J30-J99; K00-K31; K35-K38; K40-K93; L00-L99; M00-M99; N00-N99; R00-R94
External causes (EXT)	S00-T99; V01-V99; W00-W99; X00-X99; Y00-Y98
Unknown	R95-R99

6 References

1. Andersen, P. K., Canudas-Romo, V. & Keiding, N. Cause-specific measures of life years lost. *Demographic Research* **29**, 1127-1152 (2013).
2. Andreev, E. M. & Shkolnikov, V. M. Spreadsheet for calculation of confidence limits for any life table or healthy-life table quantity. *Rostock: Max Planck Institute for Demographic Research (MPIDR Technical Report* **5**, (2010).
3. Canudas-Romo, V., Adair, T. & Mazzuco, S. Reflection on modern methods: cause of death decomposition of cohort survival comparisons. *Int J Epidemiol* **49**, 1712-1718 (2020).
4. Byass, P. *et al.* An integrated approach to processing WHO-2016 verbal autopsy data: the InterVA-5 model. *BMC Medicine* **17**, 102 (2019).
